# A new species of *Centris* ( *Centris*) (Fabricius) from northeastern Brazil, with taxonomic notes on *C*. ( *C*.) *pulchra* Moure, Oliveira & Viana (Hymenoptera, Apidae)

**DOI:** 10.3897/zookeys.255.4303

**Published:** 2012-12-27

**Authors:** Thiago Mahlmann, Favízia Freitas de Oliveira

**Affiliations:** 1 Laboratório de Bionomia, Biogeografia e Sistemática de Insetos (BIOSIS), Departamento de Zoologia, Instituto de Biologia, Universidade Federal da Bahia, Rua Barão de Jeremoabo, s/n, Campus Universitário de Ondina, Salvador, CEP 40170-115, Bahia, Brasil

**Keywords:** Anthophila, Apoidea, bees, Centridini, Neotropical, taxonomy, Apinae

## Abstract

We describe a new species of the bee genus *Centris*, *Centris (Centris) byrsonimae* Mahlmann & Oliveira **sp. n.**, whose name has appeared as a *nomen nudum* in the literature since 1985. Further, a new species group of *Centris* s.str. is proposed, the *pulchra* group, based on morphological characters, which comprises the species *Centris pulchra* Moure, Oliveira & Viana, 2003 and *Centris byrsonimae* **sp. n.**.Based on information from specimen labels studied and data from the literature, a list of plant species visited by the *pulchra* group is presented. The male genitalia and hidden metasomal sterna 7 and 8 of *Centris pulchra* are described for the first time. Typographic errors pertaining to the paratype labels reported in the original description of *Centris pulchra* are corrected. One female paratype of *Centris pulchra* is designated herein as a paratype of *Centris byrsonimae* **sp. n.** An updated list of species of *Centris* s.str. from northeastern Brazil is provided including references about geographic distributions as well as an identification key to the *pulchra* species group.

## Introduction

The bee tribe Centridini (Apidae: Apinae) contains numerous robust, large to moderate-sized species, most of which are Neotropical and collect floral oils, principally from Malpighiaceae and Krameriaceae (Ayala 2002). A subgeneric classification based on a cladistic analysis was proposed by Ayala (1998), who recognized 12 subgenera in *Centris* Fabricius, two of which were described subsequently by Ayala (2002). According to Moure et al. (2012), the subgenus *Centris* is one of the most diverse containing 34 described species. According to Michener (2007), this subgenus occurs from Baja California, Mexico and southern Arizona to southern Florida, USA, and the Bahamas, and south through the Antilles and the continental tropics to Santa Catarina, Brazil. Only one species of the subgenus is exclusive to the Nearctic region, while six occur in both the Nearctic and Neotropical regions, and the remaining 27 species are exclusively distributed in the neotropics (Moure et al. 2012). In northeastern Brazilian nine nominal valid species are known for the subgenus (Table 1).

**Table 1.** List of species of *Centris (Centris)* occurring in northeastern Brazil, including the states where they have been recorded.

**Species**	**Stat^e^†**	**Reference**
*Centris aenea* Lepeletier, 1841	MA	Moure et al. (2012)
	CE	Moure et al. (2012)
	RN	Moure et al. (2012); Vivallo and Zanella (2012)^‡^
	PB	Vivallo and Zanella (2012)^‡^
	BA	Moure et al. (2012); Vivallo and Zanella (2012)^‡^
	SE	Silveira and Mendonça (2005)
*Centris byrsonimae* Mahlmann & Oliveira sp. n.	MA	Albuquerque (1986)
	CE	Paratype label
	BA	Moure et al. (2003)^§^
*Centris caxiensis* Ducke, 1907	MA	Moure et al. (2012)
	RN	Vivallo and Zanella (2012)^‡^
	BA	Vivallo and Zanella (2012)^‡^
*Centris decolorata* Lepeletier, 1841	MA	Moure et al. (2012); Vivallo and Zanella (2012)^‡^
	BA	Moure et al. (2012)
*Centris flavifrons* (Fabricius, 1775)	MA	Albuquerque (1986)
	BA	Vivallo and Zanella (2012)^‡^
	SE	Silveira and Mendonça (2005)
*Centris nitens* Lepeletier, 1841	BA	Viana and Santos (2002)
	SE	Silveira and Mendonça (2005)
*Centris pulchra* Moure, Oliveira & Viana, 2003	BA	Viana (1999); Moure et al. (2003)^§^; Moure et al. (2012)
*Centris spilopoda* Moure, 1969	MA	Albuquerque (1986)
	CE	Vivallo and Zanella (2012)^‡^
	BA	Vivallo and Zanella (2012)^‡^
*Centris varia* (Erichson, 1849)	MA	Moure et al. (2012)
	RN	Vivallo and Zanella (2012)^‡^
	BA	Vivallo and Zanella (2012)^‡^

^†^States in North-South order / MA: Maranhão, CE: Ceará, RN: Rio Grande do Norte, PB: Paraíba, BA: Bahia, SE: Sergipe.^‡^Records compiled by Vivallo and Zanella (2012).^§^Cited as *Centris pulchra* [*partim*].

The present paper describes a new species of *Centris* s.str. from northeastern Brazil. *Centris byrsonimae* sp. n., whose epithet has appeared as a *nomen nudum* in the literature since 1985, is described and figured herein, finally validating the name for this species. Based on morphological characters, a new species group of *Centris* s.str. is proposed, the *pulchra* group, which currently comprises *Centris pulchra* and *Centris byrsonimae* sp. n. In addition, we provide an identification key to this group. A list of plant species visited by the *pulchra* group is provided based on information from specimen labels and a survey of the literature. The male terminalia of *Centris pulchra* is described for the first time. Typographic errors reported from the paratype labels of *Centris pulchra* in its original description were discovered during the course of this work and herein they are corrected. One female paratype of *Centris pulchra* is designated as a paratype of *Centris byrsonimae* sp. n.

## Methods

We examined 30 paratypes (24♀♀, 6♂♂) and additional specimens of *Centris pulchra* deposited in the Entomological Collection, Zoological Museum, Federal University of Bahia (MZUFBA), Salvador, Bahia, Brazil. In addition we studied the specimen originally examined by Pe. Moure in 1985 and labeled by him as holotype of *Centris byrsonimae* (DZUP: Federal University of Paraná, Curitiba, Paraná, Brazil) but until now undescribed. Additional material was borrowed from the Entomological Collection of the Studies on Bees Laboratory (LEACOL), Federal University of Maranhão, São Luís, Maranhão, Brazil. General morphological terminology follows Engel (2001) and Michener (2007), with the standard abbreviations as follows: F1, F2, etc.– antennal flagellomeres; T1 to T7 and S1 to S8 – metasomal terga and sterna, respectively. In addition we have used the abbreviation SL for scape length. The upper and lower interocular distances were measured using the shortest distance between the compound eyes in frontal view. The mandibular teeth were numbered from the apex to the base of the mandible. All measurements are given in millimeters (mm). Label information from separate labels are segregared by double slashes,“//”. Typographic errors from paratype labels as reported in the original description of *Centris pulchra* were corrected and the corrections identified with brackets, “[ ]”. Floral records for *pulchra* species group were based on information from specimen labels and the literature. Additionally, a label reporting the host plant of the holotype of *Centris byrsonimae* sp. n. was included. Photomicrographs were prepared using a Leica M165C stereomicroscope coupled with a Leica DFC295 and a Leica Application Suite V4.1 Interactive Measurements, Montage.

## Systematics

### Tribe Centridini Cockerell & Cockerell, 1901

Genus *Centris* Fabricius, 1804

Subgenus *Centris* Fabricius, 1804

#### Pulchra

##### Diagnosis.

Herein we propose the *pulchra* species group as a distinct lineage within *Centris* s.str., comprising for the moment two species: *Centris pulchra* and *Centris byrsonimae* sp. n. This species group is characterized by the clypeus largely yellow with two longitudinal dark brown streaks located in the upper half; a narrow band of whitish and dense bristles bordering the posterior border of T2–T4, extended to the sides and narrowed in the middle, but not interrupted; by the maxillary palpus reduced to four palpomeres; the mandibles with five teeth, the most basal substantially smaller and located in the lower inner edge of the mandible; and the secondary basitibial plate yellow, but only slightly elevated (merely inflated), not projecting over the basal plate.

**Comments.** In the identification keys of Ayala (in Michener 2007) and Silveira et al. (2002), the species of the *pulchra* group do not fit well into *Centris* s.str. based on four characters: (1) the maxillary palpus is 5- or 6-segmented, (2) the mandible 3- or 4-toothed, (3) the basitibial plate with a defined secondary plate with sharp projecting margins, and (4) the clypeus with yellow or white markings in the form of an inverted T or Y. Although the *pulchra* group differs in these four characters and indicated in the key as diagnostic for the subgenus *Centris*, *Centris pulchra* and *Centris byrsonimae* sp. n. can be retained there based on the unique character of the subgenus: the long, slender, apical projection of the male gonocoxite, extending parallel to the gonostylus (Michener 2007); this gonocoxal projection bearing giant branched setae. In regard to parasitism by *Mesonychium asteria* (Smith, 1854) (Apinae: Ericrocidini) in nests of *Centris pulchra* reported by M.C. Ramos (pers. comm. in Rocha-Filho et al. 2009), this was probably a mistake. According to Marina C. Ramos (pers. comm. 2012) the parasitism observed was of an unidentified species of *Mesoplia* Lepeletier de Saint Fargeau (Apinae: Ericrocidini) in nests of *Centris byrsonimae* sp. n. She observed adults of *Mesoplia* sp. emerging from brood cells of *Centris byrsonimae* sp. n. nesting in the ground.

#### Centris (Centris) byrsonimae

Mahlmann & Oliveira sp. n.

urn:lsid:zoobank.org:act:D05DE772-DC5B-494D-8DA2-B46290F2F52F

http://species-id.net/wiki/Centris_byrsonimae

Figures 1 12 19–22

Centris (Paremisia) byrsonimae ; Albuquerque 1986: 119 [*nomen nudum*]; Albuquerque and Mendonça 1996: 47, 49, 53 [*nomen nudum*].
Centris byrsonimae ; Ribeiro et al. 2008: 167 [*nomen nudum*]; Rêgo 2008: 23 [*nomen nudum*, photo on Murici flower].
Centris aff. *pulchra*; Rosa 2009: 65.

##### Holotype.

♀, São Luís, MA, Brasil, 15.ix.1984, Albuquerque *Leg*. // Coletada na flor, *Byrsonima crassifolia*: Malpighiaceae // Holótipo // *Centris byrsonimae* Moure, 1985, Holotype // *Centris birsonimae* Moure, 1986 // Hr 15:00–16:00 // Holotype ♀ *Centris byrsonimae* Mahlmann & Oliveira, 2012. The specimen is in excellent condition and is deposited in DZUP.

##### Allotype.

♂, Alótipo // Barreirinhas, MA, Brasil, 09.viii.1991, Brito & Mendonça // Pl. N.017, 10–11h // *Centris* sp.2,* C. byrsoni*. Mou. // Allotype ♂ *Centris byrsonimae* Mahlmann & Oliveira, 2012. The specimen is deposited in MZUFBA.

##### Paratypes.

(13♀♀, 3♂♂): Parátipo // Barreirinhas, MA, Brasil, 19.xi.1991, Brito & Mendonça // Pl. N.006, 9–10h // *Centris byrsonimae* // Coletada em *Banisteriopsis* sp.: Malpighiaceae (**1**♀, LEACOL); Parátipo // São Luis, MA, Brasil, 21.x.1984, Albuquerque *Leg*. // Hr. 10:00–11:00 // Moure, 1985, Paratype // Coletada em *Byrsonima crassifolia* (L.) Kunth: Malpighiaceae (**1**♀, LEACOL); Parátipo // São Luis, MA, Brasil, 21.x.1984, Albuquerque *Leg*. // Hr. 7:00–8:00 // Paratype // Coletada em *Byrsonima crassifolia* (L.) Kunth: Malpighiaceae (**1**♀, LEACOL); Parátipo // São Luis, MA, Brasil, 19.viii.1984, Albuquerque *Leg*. // Hr. 10:00–11:00 // *Centris byrsonimae //* Moure, 1985, Paratype // Coletada em *Byrsonima crassifolia* (L.) Kunth: Malpighiaceae (**1**♀, MZUFBA); Parátipo // Ilha de Curupu, MA, Br., 14.viii.1999, Sousa & Martins *Leg*. // Pl.N.006, Hr. 13:14, 62 (**1**♀, LEACOL); Parátipo // Urbano Santos, MA, Brasil, 03°12'28"S, 43°24'12"W, 27.viii.2005, 6:00–7:00, Mendes FN, *Leg*. // 795 // Coletada em *Byrsonima umbellata* Mart. ex A.Juss.: Malpighiaceae (**1**♀, LEACOL); Parátipo // Urbano Santos, MA, Brasil, 03°12'28"S, 43°24'12"W, 27.viii.2005, 7:00–8:00, Mendes FN, *Leg*. // 800 // Coletada em *Byrsonima umbellata* Mart. ex A.Juss.: Malpighiaceae (**1**♀, LEACOL); Parátipo // Urbano Santos, MA, Brasil, 03°12'28"S, 43°24'12"W, 10.ix.2005, 9:00–10:00, Mendes FN, *Leg*. // 849 // Coletada em *Byrsonima umbellata* Mart. ex A.Juss.: Malpighiaceae (**1**♀, MZUFBA); Parátipo // Barreirinhas, MA, Brasil, 09.viii.1991, Brito & Mendonça // Pl. N.017, 10–11h // *Centris* sp.2 // *Centris byrsonimae* Moure, MS, Det. Camargo, 1992 (**1**♂, LEACOL); Parátipo // Barreirinhas, MA, Brasil, 22.viii.1992, Brito & Mendonça // Pl. N.064, 11–12h (**1**♂, LEACOL); Parátipo // Barreirinhas, MA, Brasil, 21.viii.1992, Brito & Mendonça // Pl. N.064, 12–13h (**1**♂, MZUFBA); Parátipo // Brasil, Ceará, Horizonte, 10.ix.2011, Rede Entomológica, 9h, P. Andrade *Leg*. // Coletada na flor, *Anacardium occidentale* L.: Anacardiaceae // 544 (**1**♀, MZUFBA); idem // 546 (**1**♀, MZUFBA); Parátipo // Brasil, Ceará, São Gonçalo do Amarante, Praia do Pecém, xi.2008, T. Mahlmann & Y. Antonini *Leg*. // Favízia 001153 (**1**♀, Snow Entomological Collections, University of Kansas Natural History Museum-SEMC, Lawrence, Kansas, USA); Parátipo // Brasil, Bahia, Camaçari, 04.xi.2008, J.F. Rosa e equipe *leg*.//987, Litoral Norte, Ponto 1, 04.xi.2008, 9:20–10:20, *Byrsonima sericea*, Rosa, JF; Monteiro, D; Silva, MD; Oliveira JFL *leg*. // sp.11 (**1**♀, MZUFBA); Parátipo // Brasil, Bahia, Salvador, Abaeté, 05.xi.1996, 11:15hs, B.F. Viana *Leg*. // Coletada na flor: *Byrsonima teopteridifolia* Juss [*Byrsonima microphylla* A.Juss.], Planta Nº1 // Abaeté, SSa, Ba, 5.xi.1996, N, pl:01, 11:15 // 21 // 2330 // Paratype ♀, *Centris pulchra* Moure, Oliveira & Viana, 2003 (**1**♀, MZUFBA). All specimens with the same Paratype yellow labels: Paratype *Centris byrsonimae* Mahlmann & Oliveira, 2012.

##### Diagnosis.

This species is quite similar to *Centris (Centris) pulchra* but differs by its smaller body size (ca. 10.6); the largely honey-brown integument (Figs. 2, 7); metallic blue iridescence almost imperceptible on metasomal terga; T1–T5 interlay covered by pale pubescence including the yellowish pubescence, largely golden on apical margin of T5 (Fig. 7); inner surface of probasitarsus with the combs for collecting floral oil, on distal half of posterior margin with three distinct strongly spatulate setae, curved on the inner surface and wider on its apex (Fig. 6); and the gonocoxal projection covered by dense, giant branched setae on the inner surface, forming a distinct fringe of plumose hairs longer than the gonocoxal projection itself (Figs. 19–21).

##### Description.

**Figures 1–8.**
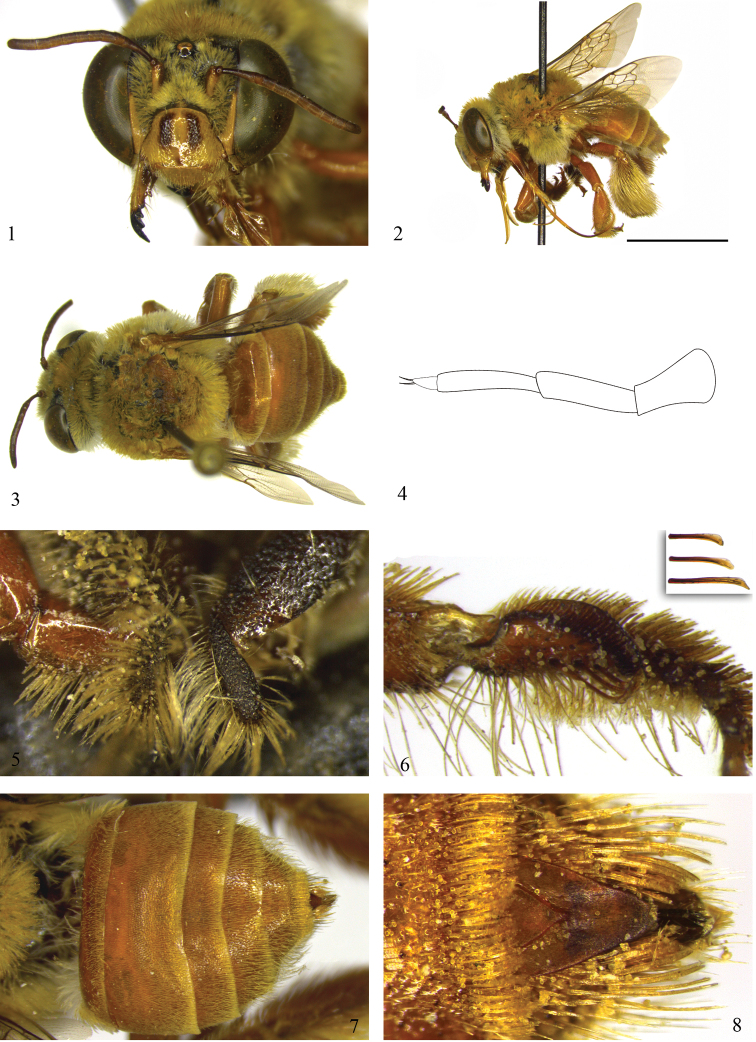
Photomicrographs and illustration of female of *Centris (Centris) byrsonimae* Mahlmann & Oliveira sp. n./ **1–3** and **7–8** holotype (DZUP) **5–6** paratype (MZUFBA). **1** Facial aspect **2** Lateral habitus, scale 0.5 cm **3** Dorsal habitus **4** Maxillary palpus **5** Procoxae **6** Probasitarsus with combs for collecting floral oil **7** Metasoma **8** Pygidial plate.

♀: *Structure*: Total body length 10.6; forewing length 7.1; head length 2.9, width 4.4; clypeus length 1.3, width 1.8; labrum length 0.7, width 1.2; scape length 0.6; F1 length 0.7; F2 length 0.2; F3 length 0.3; diameter of the anterior ocellus 0.3; ocellocular distance 0.4 (1.1x lateral ocellar diameter); upper interocular distance 2.1; lower interocular distance 2.0; metasomal width 4.2 (measured on T2). Clypeus convex with a smooth bottleneck in upper third from tentorial foveae; labrum semicircular. Maxillary palpus with four palpomeres (Fig. 4), 1st palpomere bulged and 0.5× shorter than subsequent two palpomeres, 4th palpomere much shorter than others (0.3× smaller than 2nd and 3rd). Mandibles with four normal teeth and one internal basal denticle separated from 3rd by a distance equivalent to 2× its length (Fig. 1). Posterior margin of procoxae with a broad process (Fig. 5), projected ventrally over mesepisternum and covered with long plumose pubescence. Basitibial plate with distinct secondary plate, primary plate concave and oval, secondary plate only slightly elevated but not projecting over primary plate; upper pygidial plate relatively long and narrow with its apex away from apex of lower plate by approximately 0.8× SL; inferior plate gently depressed from sides to middle and flat on apical third, ending in a point (Fig. 8).

*Coloration*: Integument predominantly honey brown with metallic blue highlights almost imperceptible on metasoma (Fig. 7); head and mesosoma dark brown, mesoscutellum brownish. Yellow marks: clypeus, except two divergent wide dark brown stripes, located on upper half of clypeus and above separated from each other by distance equivalent to lateral ocellar diameter; labrum; basal 2/3 of mandibles; malar area; very short narrow triangle on supraclypeal area; paraocular stripe ventrally wider, widest at level of tentorial fovea, narrowing upward and gradually ending at median level of compound eyes; wide stripe on lateroposterior surface of scape; small yellow marks on base of tibiae, most developed on secondary basitibial plate on metatibia; on protibia yellow marks followed by long dorsolateral stripe. Tegulae translucent honey colored; wing membrane slightly yellowish, with little brownish streak in basal third of marginal cell, with very thin arched transverse vein on apical third of first submarginal cell; brown venation slightly yellowish at pterostigma and base of wings; primary basitibial plate honey translucent; metallic blue reflections almost imperceptible on T2–T5.

*Pubescence*: In general pale and yellowish, with mostly whitish hairs as follows: lower surface of face and gena, ventral portion of mesepisterna, metepisterna, propodeum, base of metasoma and sterna; reddish bristles only on inner surface of tarsi, mostly visible on basitarsi; mesepisterna with plumose hairs restricted to upper half, dorsoventral surface covered by simple setae more widely spaced; lower 2/3 of ventral surface of procoxae covered by extremely thick, curved, yellow-whitish setae, decreasing in length toward posterior border, upper portion of procoxae covered by plumose hairs; outer surface of mesocoxae covered by simple bristles, upper margins with plumose hairs 0.5x smaller; probasitarsus with long line of bristles (2× SL) on outer dorsolateral surface, shortening toward apex and interspersed by a dense band of short plumose hairs, inner surface with few short bristles sparsely distributed; inner surface of probasitarsus, on distal half of posterior margin, with combs for collecting floral oils with three distinct and strongly spatulate setae, curved toward inner surface and wider at apex (Fig. 6); outer surface of mesobasitarsus with dense, short, plumose pubescence interspersed with simple bristles longer and sparser; metatibiae and metabasitarsus with thicker, milky white bristles; T2–T4 entirely covered by pale yellow, short and sparse setae, longer and denser on sides of terga and near posterior border, contrasting with hairiness of discal terga but not forming distinct band of yellowish bristles (Fig. 7); apical margin of T5 with long fringe of golden branched bristles (0.5× SL); S2–S5 with dense apical fringe, with hairs of central stripe slightly longer; fringe of S5 curved and with rachis relatively longer.

*Sculpturing*: Dense but of moderate size: on vertex spacing 1/2 of puncture width, with cariniform spaces between punctures, slightly smoother on supraclypeal area; midline of clypeus with integument smooth and polished, interspersed by very few punctures; punctures most evident on the side slopes of clypeus and labrum. Punctures obscured by pilosity on mesosoma and metasoma, mostly fine and spaced on sides of propodeum, and finer and piligerous on terga.

♂: *Structure*: Total body length 10.8; forewing length 7.7; head length 2.7, width 4.1; clypeus length 1.2, width 1.7; labrum length 0.8, width 1.2; scape length 0.6; F1 length 0.7; F2 length 0.2; F3 length 0.3; diameter of the anterior ocellus 0.3; ocellocular distance 0.3 (1.3× lateral ocellar diameter); upper interocular distance 2.0; lower interocular distance 2.3; metasoma width 4.2 (measured on T2). Similar to female except as follows: mandibles with three normal teeth and one basal denticle on inner margin; basitibial and pygidial plates absent. S7 and S8 as in figure 22: S7 about as long as wide as measured at base, approximately 2/3 of S8 length and covered by setae on its lateral areas and apex; apical margin of S7 indented; median projection of S8 slightly uniformly tapering to apex; dorsal surface and apical 1/3 of S8 covered by long branched setae, apex covered by simple, shorter erect setae. Genitalia as in figures 19–21: dorsal surface of gonocoxite with small basal edge, internal surface covered by long, dense pilosity; gonocoxal projection covered by dense, long branched setae on inside surface forming distinct fringe of plumose hairs longer than gonocoxal projection itself; gonostylus covered by short setae.

**Figures 9–12.**
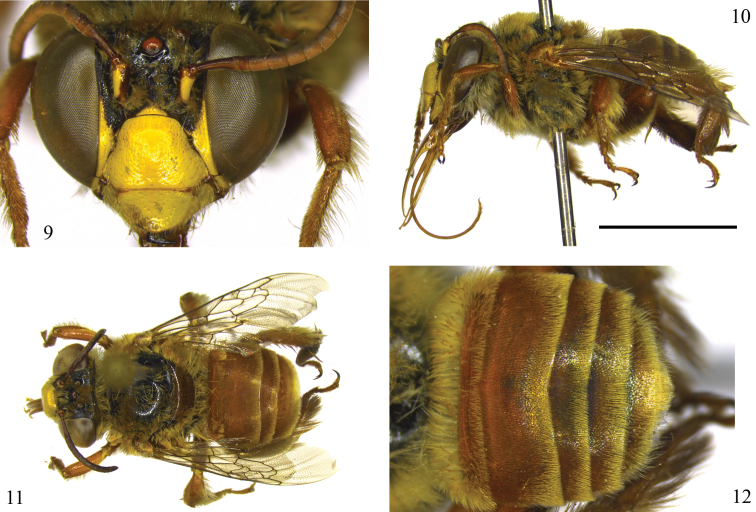
Photomicrographs of male of *Centris (Centris) byrsonimae* Mahlmann & Oliveira sp. n./ **9–11** allotype (MZUFBA) **12** paratype (MZUFBA) **9** Facial aspect **10** Lateral habitus, scale 0.5 cm **11 **Dorsal habitus **12** Metasoma.

*Coloration*: Metallic blue–greenish reflections more visible than in female at T2–T5 (Fig. 12); clypeus without two divergent dark brown stripes on upper half (Fig. 9).

*Pubescence*: Similar to female but mesepisterna fully covered by dense plumose hairs; ventral surface of pro- and mesocoxae covered by plumose hairs only, procoxae with plumose hairs markedly longer, denser and recurved; probasitarsus without long line of long bristles; inner surface of probasitarsus without combs for collecting floral oils; outer surface of mesobasitarsus without velvet, dense, short plumose pubescence; external surface of metabasitarsus covered by dark-brown setae; base of metapretarsal claws with pair of thin, simple setae almost as long as pretarsal claws; pilosity of T1 denser and longer than remaining terga; T2–T5 with band of yellowish setae more distinctive and wider than in female (Fig. 12); margin of T5 without long fringe of golden branched bristles.

*Sculpturing*: Midline of clypeus with integument mostly smooth and polished, interspersed by very few punctures.

##### Etymology.

The specific epithet was originally given by Dr. Jesus S. Moure in 1985 when he identified the material collected by Dra. Patrícia Maia C. de Albuquerque for her dissertation, using the generic name of the most common host plant for this bee species (*Byrsonima crassifolia* L. Rich: Malpighiaceae). Unfortunately, while Moure placed the name on labels of specimens he never published a description of the species or put into writing those characters from which he based his conclusions. In 2003, after the publication of *Centris pulchra*, Moure invited the second author of the present paper to describe with him this new species after a restudy of all paratypes of *Centris pulchra* and other specimens from Maranhão deposited in LEACOL, including the male specimens, are re-examined. However, Moure died in June 2010 before the paper was completed. Thus, despite the fact that he recognized the novelty of the species, his name is not included as a coauthor given that he did not review or approve any version of this manuscript. Nonetheless, we retained the name he intended as well as the specimen he selected as the holotype. Such an epithet also seems to be a good choice given that it has been used before in the literature.

##### Comments.

Although *Centris byrsonimae* sp. n.is quite similar to *Centris pulchra*, it can be differentiated from that species by the integument predominantly honey-brown (mostly dark brown in *Centris pulchra*); *Centris byrsonimae* with metallic blue highlights almost imperceptible on metasomal terga, marked in *Centris pulchra*. The pubescence in general is yellowish, especially on the disc of the terga and the apex of T5, with an apical fringe of golden bristles at the apex of T5; in *Centris byrsonimae* sp. n. T2–T4 are entirely covered by pale, short yellow and sparse setae on the disk of the terga but not forming a distinct band of bristles on the posterior border, while in *Centris pulchra* the discs of T2–T4 are covered by dark-brown setae contrasting with the narrow and dense bands of whitish bristles bordering the posterior edge of the terga extending to the sides and narrowed in the middle, but not interrupted, and contrasting also with the hairiness of the disc of the terga. On the mesepisterna of *Centris byrsonimae* sp. n.the plumose hairs are restricted to the upper half, while in *Centris pulchra* they are distributed on the dorsoventral surface; the lower 2/3 of the ventral surface of the procoxae are covered by extremely thick, curved yellow-whitish setae (the upper portion covered by plumose hairs) in *Centris byrsonimae* sp. n.while in *Centris pulchra* this structure has only plumose hairs; the inner surface of the probasitarsus with secondary combs for collecting floral oils is modified, with three distinct, strongly spatulate setae in *Centris byrsonimae* sp. n.while in *Centris pulchra* these setae are thick but not modified (Figs. 6, 16); the pygidial plate of both species is doubled but in *Centris byrsonimae* sp. n.the upper plate is relatively longer (Fig. 8); the lower plate of the pygidial plate is slightly depressed from the sides to the middle and flat on the apical third contrasting with *Centris pulchra* in which this depression is notably more pronounced and convex in the apical third (Fig. 18). The male of both species are quite similar but in *Centris byrsonimae* sp. n.the pubescence is markedly yellowish while in *Centris pulchra* it is whitish; the metallic highlights are most visible and bluish in *Centris pulchra* and almost imperceptible and greenish in *Centris byrsonimae* sp. n.; the base of the metapretarsal claws have a pair of thin simple setae in *Centris byrsonimae* sp. n.while in *Centris pulchra* there is a tuft of long dense plumose hairs. In addition, the male terminalia is quite different as shown in Figures 19–26.

##### Floral records.

Table 2

**Table 2.** Floral records for *Centris pulchra* (Cp.) and *Centris byrsonimae* sp. n.(Cb.), with respect to the sex of the floral visitor recorded. Botanical classification follows that of the *Flora do Brasil* (2012).

Botanic Species	Cp.	Cb.	References	State
Anacardiaceae				
Anacardium occidentale L.	-	♀	Paratype label	CE
Fabaceae				
Chamaecrista ramosa (Vog.) I & B var. ramosa	♀	-	Moure et al. (2003)	BA
Galactia remansoana Harms	?	-	Pigozzo and Viana (2010)	BA
Mimosa xiquexiquensis Barneby	?	-	Rodarte et al. (2008)	BA
Stylosanthes viscosa (L.) Sw.	♀	-	Moure et al. (2003)	BA
Krameriaceae				
Krameria bahiana B. Simpson	♀♂	-	Moure et al. (2003)	BA
Lamiaceae				
Eriope blanchetii (Benth.) Harley	♀	-	Moure et al. (2003)	BA
Lythraceae				
Cuphea brachiata Koehne	♀♂	-	Moure et al. (2003)	BA
Malpighiaceae				
Banisteriopsis sp.	-	♀	Albuquerque 2012 (pers. comm.)	MA
Byrsonima blanchetiana Miq.	?	-	Rodarte et al. (2008)	BA
Byrsonima chrysophylla Kunth	-	?	Ribeiro et al. (2008)	BA
Byrsonima crassifolia (L.) Kunth	-	♀	Albuquerque (1986)	MA
Byrsonima microphylla A.Juss.	-	♀	Moure et al. (2003)†	BA
Byrsonima sericea DC.	-	♀	Rosa (2009)‡	BA
Byrsonima umbellata Mart. ex A. Juss.	-	♀	Albuquerque 2012 (pers. comm.)	MA
Malvaceae				
Waltheria cinerescens A.St.-Hil.	♀♂	-	Moure et al. (2003)	BA
Ochnaceae				
Ouratea rotundifolia (Gardner) Engl.	♀	-	Moure et al. (2003)	BA
Turneraceae				
Turnera calyptrocarpa Urb.	?	-	Rodarte et al. (2008)	BA

^†^Cited as *Byrsonima teopteridifolia* and *Centris pulchra* [*partim*] by Moure et al. (2003)^‡^Cited as *Centris* aff. *pulchra*

#### Centris (Centris) pulchra

Moure, Oliveira & Viana, 2003

http://species-id.net/wiki/Centris_pulchra

Figures 13–18 23–26

Centris (Centris) pulchra Moure, Oliveira & Viana, 2003 [*partim*]; Rocha-Filho et al. 2009: 301 [parasitism record, in error]; Pigozzo and Viana 2010: 105, 109 [floral record]; Vivallo and Zanella 2012: 5, 7, 9, 11, 12 [key, *partim*]; Moure et al. 2012 [catalogue].
Centris (Paremisia) pulchra ; Viana 1999: 638 [checklist, *nomen nudum*]; Viana and Santos 2002: 147 [*nomen nudum*, *partim*]; Viana and Kleinert 2005: 7 [checklist, *partim*]; Viana and Kleinert 2006: 58 [floral records, *partim*]; Rodarte et al. 2008: 307 [floral records].

##### Remarks.

One female specimen originally designated by Moure et al. (2003) as paratype of *Centris pulchra* is actually an individual of the species described above as *Centris byrsonimae* sp. n.and is selected as a paratype of that species (Parátipo ♀ // Brasil, Bahia, Salvador, Abaeté, 05.xi.1996, 11:15hs, B.F. Viana *Leg*. // Coletada na flor: *Byrsonima teopteridifolia* Juss, Planta Nº1 // Abaeté, SSa, Ba, 5.xi.1996, N, pl:01, 11:15 // 21 // 2330 // Paratype ♀, *Centris pulchra* Moure, Oliveira & Viana, 2003). For this female paratype a mistake was made in reporting the identification of the host plant in the original description. The plant identification was subsequently corrected by the specialist who first identified the botanical species considered presently as *Byrsonima microphylla* A.Juss. (F.O. da Silva and B.F Viana 2012, pers. comm.). Moure et al. (2003) listed six male paratypes, probably a typographic mistake due the fact that one specimen with the label (Parátipo // Brasil, Bahia, Salvador, Abaeté, 11.ix.1996, 13:35hs, B.F. Viana *Leg*. // Coletada na flor: *Waltheria cinerescens* St. Hil, Planta Nº22 // Abaeté, SSa, Ba, 11.ix.1996, N, pl:22, 13:35 // 21, ♂ // 2319 // Paratype ♂, *Centris pulchra* Moure, Oliveira & Viana, 2003) is a male, giving a total of 22♀♀ and 7♂♂ paratypes for *Centris pulchra* in MZUFBA. Two other typographic mistakes were observed about the labels as recorded in the original description of *Centris pulchra*. The data for these two paratypes are corrected here: (Parátipo // Brasil, Bahia, Salvador, Abaeté, 01.ix.1996, 12:20hs, B.F. Viana *Leg*. // Coletada na flor: *Eriope blanchetii* (Benth) Harley, Planta Nº10 // Abaeté, SSa, Ba, 01.ix.1996, N, pl:10, 12:20 // 21 // 2328 // Paratype ♀, *Centris pulchra* Moure, Oliveira & Viana, 2003) and (Parátipo // Brasil, Bahia, Salvador, Abaeté, 13.iv.1996, 14:20hs, B.F. Viana *Leg*. // Coletada na flor: *Cuphea branchiata* Koehne, Planta Nº3 // Abaeté, SSa, Ba, 13.iv.1996, N, pl:03, 14:20 // 21, ♂ // 2317 // Paratype ♂, *Centris pulchra* Moure, Oliveira & Viana, 2003).

##### Diagnosis.

Total body length 13.9. Integument brown-blackish, except legs and metasoma ferruginous; metallic blue highlights on T2 – T5, mostly marked on T5 (Fig. 17); distal edge of terga with a band of whitish setae; most part of basal area of T2–T4 with black bristles. Inner surface of probasitarsus with combs for collecting floral oil, on distal half of posterior margin, with setae unmodified (Fig. 16). Male genitalia with pubescence relatively spaced, apical projection of gonocoxite with a fringe of short, unbranched bristles, about 1/2 length of projection itself (23–25).

Male S7, S8 and genitalia as in figures 23–26: S7 notably wider than long, approximately 1/5 of length of S8; edge of S7 strongly angular, forming two distinct lobes, these covered with erect bristles; median projection of S8 with a strong median strangulation, apical 1/3 enlarged; dorsal surface of S8 covered by erect bristles, apical 1/3 covered by longer branched bristles curved laterally, apex with shorter simple erect bristles. Dorsal surface of gonocoxite with a large basal edge strongly keeled, internal border covered by short dense pilosity; apical projection of gonocoxite relatively short, its apex distant from gonostylus apex and covered internally by a fringe of simple setae shorter than projection; gonostylus covered by short bristles sparsely distributed (Figures 23–25).

**Figures 13–18.**
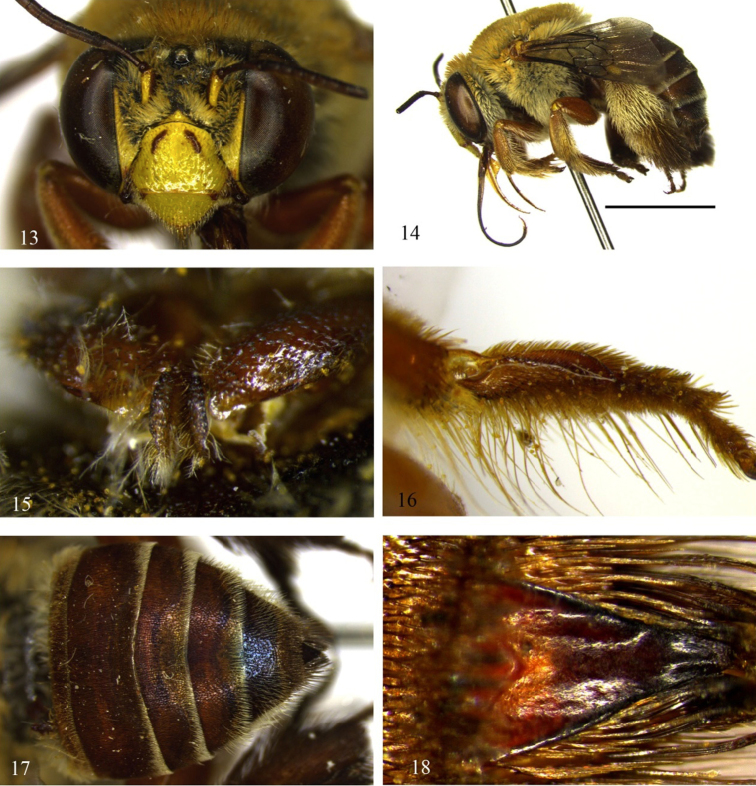
Photomicrographs of female of *Centris (Centris) pulchra* Moure, Oliveira & Viana, 2003/ **13–14** and **16–18** paratype (MZUFBA) **15** additional material (MZUFBA). **13** Facial aspect **14** Lateral habitus, scale 0.5 cm **15** Procoxae**16** Probasitarsus with combs for collecting floral oil**17** Metasoma **18** Pygidial plate.

**Figures 19–26.**
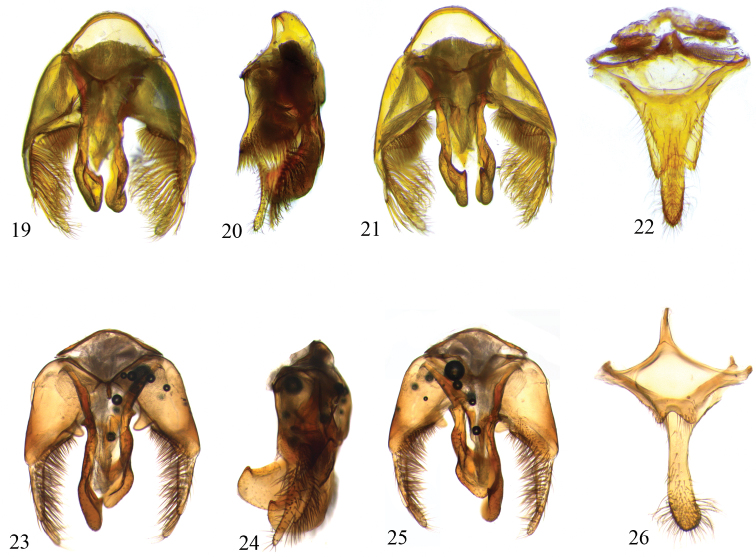
Photomicrographs of male terminalia of *Centris (Centris) byrsonimae* Mahlmann & Oliveira sp. n.(19–22, allotype/MZUFBA) and *Centris (Centris) pulchra* Moure, Oliveira & Viana 2003 (23–26, additional material/MZUFBA). **19, 23** Genital capsule, dorsal view **20, 24** Genital capsule, lateral view **21, 25** Genital capsule, ventral view **22, 26** Seventh and eighth metasomal sternum.

##### Floral records. 

Table 2

### Key to species of the *pulchra* species group

**Table:** 

1	Female	2
-	Male	3
2(1)	Integument predominantly dark brown to black (Figs. 14, 17); inferior 2/3 of ventral surface of procoxae with tufts of long plumose hairs, interspersed by very thin, long, simple hair, superior portion of coxae practically glabrous; discs of T2–T4 covered by dark brown to black setae, contrasting with whitish fringe of hairs on posterior border; probasitarsus with secondary unmodified combs for collecting floral oil (Fig. 16)	*Centris pulchra* Moure et al.
-	Integument predominantly honey brown (Figs. 2, 7); inferior 2/3 of ventral surface of procoxae covered by extremely thick, curved, yellow-whitish setae, decreasing in length toward posterior border, superior portion of coxae covered by plumose hairs; T2–T4 entirely covered by yellowish short and sparse setae most longer and denser on sides of terga and near posterior border, contrasting with hairiness of disc of terga but not forming a distinct band of whitish bristles; probasitarsus with secondary combs for collecting floral oil modified, with three distinct strongly spatulate setae, curved toward inner surface and with enlarged apex (Fig. 6)	*Centris byrsonimae* sp. n.
3(1)	Base of metapretarsal claws with tuft of long, dense plumose hairs; apical projection of gonocoxite covered internally by a fringe of simple setae shorter than projection (Figs. 23–25)	*Centris pulchra* Moure et al.
-	Base of metapretarsal claws with a pair of long thin simple setae; apical projection of gonocoxite covered internally by a very distinct fringe of branched setae longer than projection (Figs. 19–21)	*Centris byrsonimae* sp. n.

## Supplementary Material

XML Treatment for Pulchra

XML Treatment for Centris (Centris) byrsonimae

XML Treatment for Centris (Centris) pulchra

## References

[B1] Albuquerque PMC (1986) O “Murici” (*Byrsonimacrassifolia*, Malpighiaceae) - aspectos históricos, fenologia e abelhas polinizadoras. Graduation thesis, São Luis, Maranhão, Brasil: Universidade Federal do Maranhão.

[B2] Albuquerque PMC Mendonça JAC (1996) Anthophoridae (Hymenoptera; Apoidea) e flora associada em uma formação de cerra do no município de Barreirinhas, MA, Brasil. Acta Amazonica 26 (112): 45-54.

[B3] Ayala R (1998) Sistemática supra especifica de las abejas de la tribu Centridini (Hymenoptera: Apoidea). PhD thesis, Universidad Nacional Autonoma de Mexico.

[B4] Ayala R (2002) Two new subgenera of bees in the genus *Centris* (Hymenoptera: Apidae). Scientific Papers, Natural History Museum, University of Kansas 25: 1-8.

[B5] Cockerell TDA Cockerell WP (1901) VIII.–Contributions from the New Mexico biological station–IX. On certain genera of bees. Annals and Magazine of Natural History, Series 7, 7: 46–50. doi: 10.1080/00222930108678438

[B6] Engel MS (2001) A monograph of the Baltic amber bees and evolution of the Apoidea (Hymenoptera). Bulletin of the American Museum of Natural History 259: 1-192. doi: 10.1206/0003-0090(2001)259<0001:AMOTBA>2.0.CO;2

[B7] Flora do Brasil (2012) Lista de Espécies da Flora do Brasil. http://floradobrasil.jbrj.gov.br/2012/ [Accessed Oct/04/2012]

[B8] Fabricius JC (1804) Systema Piezatorum secundum ordines, genera, species, adjectis synonymis, locis, observationibus, descriptionibus. Brunsvigae: Reichard, 439 pp.

[B9] Michener CD (2007) The Bees of the World [2nd. Edition] Baltimore: Johns Hopkins University Press. Baltimore, 953pp.

[B10] Moure JS Melo GAR Vivallo F (2012) Centridini Cockerell & Cockerell, 1901. In: Moure JS, Urban D, Melo GAR (Eds.) Catalogue of Bees (Hymenoptera, Apoidea) in the Neotropical Region, online version. http://www.moure.cria.org.br/catalogue [Accessed Aug/13/2012]

[B11] Moure JS Oliveira FF Viana BF (2003) Descrição de uma espécie nova de *Centris* Fabricius da Bahia, Brasil (Hymenoptera, Apoidea). Revista Brasileira de Entomologia 47 (4): 569-572. doi: 10.1590/S0085-56262003000400006

[B12] Pigozzo CM Viana BF (2010) Estrutura da rede de interações entre flores e abelhas em ambiente de Caatinga. Oecologia Australis 14 (1): 100-114. doi: 10.4257/oeco.2010.1401.04

[B13] Rêgo MMC (2008) Pollinators of nance (*Byrsonima crassifolia* L. Rich, Malpighiaceae): Diversity of species, nest building and their sustainable use in agriculture in eastern Amazon. Ministry of the Environment. (Org.). Pollinators Management in Brazil, Brasilia, 22–25.

[B14] Ribeiro EKMD Rêgo MMC Machado ICS (2008) Cargas polínicas de abelhas polinizadoras de *Byrsonima chrysophylla* Kunth. (Malpighiaceae): fidelidade e fontes alternativas de recursos florais. Acta Botânica Brasilica 22 (1): 165-171. doi: 10.1590/S0102-33062008000100017

[B15] Rocha-Filho LC Morato EF Melo GAR (2009) New host records of *Aglaomelissa duckei* and a compilation of host associations of Ericrocidini bees (Hymenoptera: Apidae). Zoologia 26: 299-304.

[B16] Rodarte ATA Silva FO Viana BF (2008) A flora melitófila de uma área de dunas com vegetação de caatinga, Estado da Bahia, Nordeste do Brasil. Acta Botânica Brasílica, 22, 301–312. doi: 10.1590/S0102-33062008000200001

[B17] Rosa JF (2009) Dinâmica espacial na diversidade de abelhas Centridini: oferta de óleos florais como medida da qualidade do habitat. Master thesis, Salvador, Bahia, Brasil: Instituto de Biologia da Universidade Federal da Bahia.

[B18] Silveira FA Melo GAR Almeida EAB (2002) Abelhas Brasileiras: Sistemática e Identificação. Belo Horizonte, 253 pp.

[B19] Silveira MS Mendonça MC (2005) Diversidade de Abelhas In: Parque Nacional Serra de Itabaiana - Levantamento da Biota (Carvalho CM, Vilar JC, Coord.). Aracajú, Ibama, Biologia Geral e Experimental – UFS, 105–112.

[B20] Viana BF (1999) A comunidade de abelhas (Hymenoptera: Apoidea) das dunas interiores do Rio São Francisco, Bahia, Brasil. Anais da Sociedade Entomológica Brasileira, 28, 635–645. doi: 10.1590/S0301-80591999000400006

[B21] Viana BF Alves dos Santos I (2002) Bee diversity of the coastal sand dunes of Brazil. In: Kevan P, Imperatriz Fonseca VL (Eds) Pollinating Bees, The Conservation Link Between Agriculture and Nature, Ministry of Environment, Brasília, 135–153.

[B22] Viana BF Kleinert AMP (2005) A community of flower-visiting bees (Hymenoptera: Apoidea) in the coastal sand dunes of northeastern Brazil. Biota Neotropica 5(2). http://www.biotaneotropica.org.br/v5n2/pt/abstract?article+BN00305022005

[B23] Viana BF Kleinert AMP (2006) Structure of bee-flower system in the coastal sand dune of Abaeté, northeastern Brazil. Revista Brasileira de Entomologia 50 (1): 53-63. doi: 10.1590/S0085-56262006000100008

[B24] Vivallo F Zanella FCV (2012) A new species of *Centris* (*Paracentris*) Cameron, 1903 from northeastern Brazil, with a key for the *Centris* species of the Caatinga region (Hymenoptera: Apidae). Zootaxa 3298: 1-16.

